# The first confirmed human case of rabies, Timor-Leste, 2024

**DOI:** 10.2807/1560-7917.ES.2024.29.18.2400241

**Published:** 2024-05-02

**Authors:** Marcelo Amaral Mali, Filipe de Neri Machado, Filomeno Pinto Moniz, Frederico Bosco Alves dos Santos, Perpetua Ana Mery Estela Laot, Ari Jayanti Pereira Tilman, Tanizio Ebryes Florindo, Cristovao de Alexandria Barros, Adriano Barbosa, Jose A Oliveira Lima, Joao Paulo Goncalves, Francisco Borges, Elisabeth Hornay, Joanico Moises, Osmenia de Jesus Neto, Liliana Varela, Agapito da Costa, Anthony DK Draper, Joshua R Francis, Merita Antonio A Monteiro

**Affiliations:** 1 Instituto Nacional de Saúde Pública de Timor-Leste, Comoro, Timor-Leste; 2 Laboratorio Nacional de Saúde, Bidau, Timor-Leste; 3 Hospital Nacional Guido Valadares, Bidau, Timor-Leste; 4 Centro da Saúde, Kiu Manteko, Oecusse, Timor-Leste; 5 Menzies School of Health Research, Bidau Lecidere, Timor-Leste; 6 National Centre for Epidemiology and Population Health, Australian National University, Canberra, Australia; 7 Centre for Disease Control, Northern Territory Government Department of Health, Darwin, Australia

**Keywords:** Rabies, Timor-Leste, Southeast Asia, Dog bite, Post-exposure prophylaxis, lyssavirus

## Abstract

In March 2024, the first ever human case of rabies, following a dog bite, was detected in Timor-Leste. This paper briefly discusses the circumstances of transmission, clinical presentation, palliative care of the case and public health measures taken. Timor-Leste was previously considered rabies-free. Any person who is bitten or scratched by an animal that could potentially transmit rabies virus (especially dogs, bats, monkeys or cats) in Timor-Leste should be assessed for consideration of provision of rabies post-exposure prophylaxis.

Rabies is a viral disease, which occurs in over 150 countries [1] and is estimated to cause 59,000 deaths each year [2]. Usually, rabies virus is transmitted to humans through the bite or a scratch of an infected animal. In low- and middle-income countries, dog bites are the primary driver of transmission to humans, but the virus can also be transmitted by other animals [3]. Rabies is nearly always fatal if symptoms develop, however, it can be prevented if post-exposure prophylaxis (PEP) consisting of rabies vaccine and rabies immunoglobulin (RIG) are administered as soon as possible after being exposed to rabies virus [4]. Here we describe a fatal case of rabies in Timor-Leste.

## Epidemiological situation of rabies on the island of Timor

Timor-Leste is a resource-limited, half-island nation with a population of 1.3 million, situated 550 km north-west of Australia [5]. The western part of the island of Timor consists of the Indonesian Province of Nusa Tenggara Timur (NTT) and the Timorese municipality of Oecusse, which is an exclave surrounded by NTT (Figure).

**Figure:**
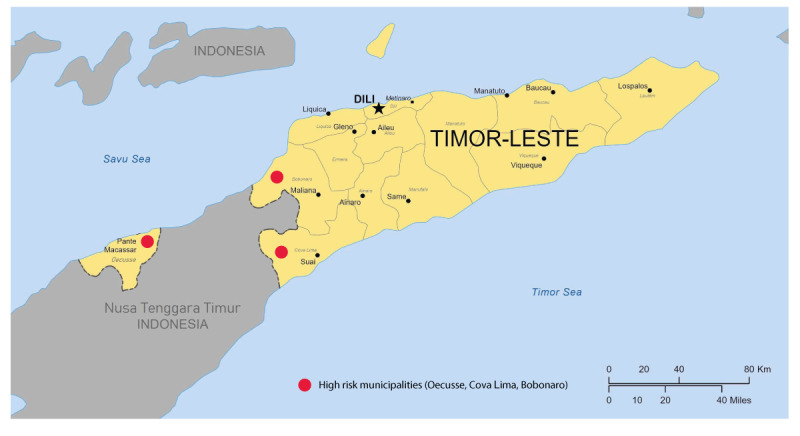
Map of Timor-Leste, showing the location of the municipality of Oecusse

Rabies is endemic in 26 provinces of Indonesia. However, the island of Timor was rabies-free until the first human rabies cases were detected in the western half of the island in May 2023 [6].

Recognising the threat of rabies entering Timor-Leste through dogs, particularly in the western municipalities of Oecusse, Cova Lima and Bobonaro, which border Indonesia, a public awareness campaign began in 2023 followed by a mass dog, cat and monkey vaccination programme which began in January 2024 [7].

## Case presentation

The case, a 19-year-old female resident of Passabe, Oecusse presented to a clinic in Pante Makassar, Oecusse on 20 March 2024. The patient was previously healthy with no previous medical history, allergies or neurological issues. She presented with 1-day history of fever, difficulty swallowing fluids, hydrophobia, photophobia, back pain, headache and neck pain. The patient appeared acutely unwell and demonstrated signs of systemic distress. At the time of the clinic visit, the patient was conscious and oriented to time and person, had a clear chest, soft abdomen without distension and no organomegaly. The patient had a blood pressure of 100/70 mmHg, respiratory rate of 18 breathes per minute, oxygen saturation of 99% and a temperature of 39.5°C (103.1°F). Rabies was immediately suspected, and the patient revealed a history of her own dog biting her hand on 26 December 2023, 12 weeks before onset of symptoms. The dog had exhibited aggressive behaviour before biting her and died the following day. The dog was not vaccinated against rabies nor was it tested. The case did not seek medical attention or PEP for rabies immediately following the dog bite. The case was reported on the day of presentation with symptoms to the Surveillance Department in the Timor-Leste Ministry of Health as a probable rabies case according to local case definitions [8,9].

The patient was transferred to the national hospital in the capital, Dili (Hospital Nacional Guido Valadares) on the evening of 21 March 2024, and a saliva sample was collected and tested by real-time PCR in the National Health Laboratory in Dili [10,11]. Rabies virus RNA was detected, and the sample was sent to the Victorian Infectious Diseases Reference Laboratory (VIDRL) in Melbourne, Australia, which confirmed the positive PCR result.

Upon admission to the hospital, the patient was moved to a quiet private room and given supportive care including intravenous fluids, analgesia, benzodiazepines and empiric antibiotics, consistent with recently developed national guidelines for clinical management of rabies. Overnight, the patient became increasingly agitated, began experiencing recurrent convulsions which gradually progressed to drowsiness and coma which resulted in death from respiratory failure on 22 March 2024, 3 days after onset of symptoms.

## Public health measures

The family of the case did not report that the dog who bit the case had bitten others. The healthcare workers treating the case all wore appropriate personal protective equipment, and there was no PEP given to healthcare workers or family of the case. There was significant local media coverage of this case which resulted in an increase in presentations of people bitten by dogs since December 2023, but who had not previously presented to medical facilities for consideration of PEP. Social media messaging promoting the public to vaccinate their dogs and present to health clinics after dog bites and scratches increased. Efforts to vaccinate the dog population in Timor-Leste, particularly in the affected municipality of Oecusse have increased. Dogs are the main focus of the animal vaccination campaign, monkeys and cats are not as common pets as dogs.

## Discussion

We report the first confirmed human rabies case in Timor-Leste, following a bite from a suspected rabid dog. Although rabies has long been endemic in Indonesia, there is a high risk of it becoming endemic in Timor-Leste. Timor-Leste can no longer be considered rabies-free. Once humans exhibit symptoms of rabies virus infection, the disease is nearly always fatal. In Timor-Leste, the options for palliative care are limited. The administration of rabies PEP directly after exposure, however, is effective at preventing rabies and devastating consequences.

Any person in Timor-Leste who is bitten by an animal that could potentially transmit the rabies virus (especially dogs, bats, monkeys or cats) should wash the wound immediately and present to a healthcare centre or hospital for consideration of provision of rabies PEP.

In Timor-Leste and particularly in remote areas, vaccine and RIG may be in limited supply. Increased awareness of the need to present for provision of vaccine and possibly RIG post rabies exposure, may pose significant challenges for stock supply. It is important that rabies vaccine and RIG stocks are maintained at active healthcare sites.

Dog vaccination programmes should continue in Timor-Leste to decrease the risk of transmission.
